# Trbp Is Required for Differentiation of Myoblasts and Normal Regeneration of Skeletal Muscle

**DOI:** 10.1371/journal.pone.0155349

**Published:** 2016-05-09

**Authors:** Jian Ding, Mao Nie, Jianming Liu, Xiaoyun Hu, Lixin Ma, Zhong-Liang Deng, Da-Zhi Wang

**Affiliations:** 1 Department of Cardiology, Boston Children's Hospital, Harvard Medical School, 320 Longwood Avenue, Boston, Massachusetts, United States of America; 2 Department of Orthopaedic Surgery, The Second Affiliated Hospital, Chongqing Medical University, Chongqing, China; 3 College of Life Sciences, Hubei University, Wuhan, Hubei, China; 4 Harvard Stem Cell Institute, Harvard University, Cambridge, Massachusetts, United States of America; Institut de Myologie, FRANCE

## Abstract

Global inactivation of Trbp, a regulator of miRNA pathways, resulted in developmental defects and postnatal lethality in mice. Recently, we showed that cardiac-specific deletion of Trbp caused heart failure. However, its functional role(s) in skeletal muscle has not been characterized. Using a conditional knockout model, we generated mice lacking Trbp in the skeletal muscle. Unexpectedly, skeletal muscle specific Trbp mutant mice appear to be phenotypically normal under normal physiological conditions. However, these mice exhibited impaired muscle regeneration and increased fibrosis in response to cardiotoxin-induced muscle injury, suggesting that Trbp is required for muscle repair. Using cultured myoblast cells we further showed that inhibition of Trbp repressed myoblast differentiation *in vitro*. The impaired myogenesis is associated with reduced expression of muscle-specific miRNAs, miR-1a and miR-133a. Together, our study demonstrated that Trbp participates in the regulation of muscle differentiation and regeneration.

## Introduction

As the most abundant tissue in vertebrates, skeletal muscle greatly contributes to the maintenance of body posture and the action of locomotion; additionally, skeletal muscle plays important roles in the modulation of systemic metabolism in animals[1, 2]. Formation and homeostasis of skeletal muscle highly depend on differentiation of myoblasts and fusion of myotubes into myofibers, which are the basic units of muscle tissue[3, 4]. The myogenic processes of creating and maintaining myofibers are essential not only for skeletal muscle development at the basal level, but also for muscle regeneration during aging and in response to injury. Myogenesis is regulated by complex mechanisms involving multiple protein factors and non-coding RNAs (ncRNAs)[1, 5]. MicroRNAs (miRNAs), a class of small ncRNAs, play critical roles in modulating gene expression. Previous studies have reported that numerous miRNAs participate in the regulation of myogenesis, muscle gene expression, and related muscle diseases[6–12].

The muscle enriched miR-1, miR-133, and miR-206 exhibit correlated expression pattern during myogenesis or in muscle regeneration. Others and we have demonstrated their regulatory roles: inhibition of miR-1, or miR-206 represses myogenesis, while overexpression of these miRNAs can promote myoblast differentiation[6, 8, 11]. miR-133 has also been reported to regulate muscle differentiation. Interestingly, whereas miR-133 was shown to promote myoblast proliferation by repressing SRF, it is also required for normal myogenic differentiation through the inhibition of uncoupling protein 2 (UCP2) [7, 10]. These miRNAs likely regulate muscle differentiation via repression of downstream protein coding genes such as Pax7, HDAC4, UCP2, Cx43 and Fstl1[7–9, 13].

The HIV TAR RNA-binding protein, Trbp, has been identified as a binding partner of Dicer and a key regulator of miRNA expression[14–16]. *In vitro* studies have shown that Trbp facilitates miRNA biogenesis[17, 18]. Numerous studies have shown that Trbp exerts its roles through miRNA-independent mechanisms[19–22]. For example, Trbp can modulate the stability of downstream tumor suppressors mRNAs in cancer cells, to promote metastasis[21]. In liver tissue, Trbp can act as a regulator of mRNA translation by repressing the Protein Kinase R (PKR) activity and modulate immuno-metabolism[22]. Recently, using genetic models, we investigated the *in vivo* functions of Trbp in cardiac muscle. We found that Trbp regulates the expression and function of cardiac-specific miR-208a, which represses the transcription factor Sox6. We further demonstrated that Trbp is required for normal contraction of the heart, at least in part, by modulating the proper expression of the fast- and slow- twitch myofiber gene program of cardiac muscle [23]. However, the biological actions of Trbp in other tissues, including skeletal muscle, remain unknown.

In this study, we design experiments to examine the function of Trbp in skeletal muscle. Interestingly, conventional knockout of Trbp in mice resulted in small body size[23, 24]. As skeletal muscle is the largest tissue and makes up a substantial part of body weight, we asked if the reduced body size was due to loss of the Trbp activity in skeletal muscle *per se*. To answer this question, we conditionally inactivated the Trbp gene in skeletal muscle by crossing the Trbp floxed mice with the myf5-Cre mice, which direct Cre recombinease in skeletal muscle specifically. Trbp mutant mice exhibited delayed muscle regeneration in response to cardiotoxin-induced muscle injury. Using a C2C12 myoblast *in vitro* system, we further showed that Trbp is required for normal myoblast differentiation. The impaired muscle differentiation in Trbp mutant mice or Trbp-knockdown cells is associated with reduced expression of miR-1a and miR-133a. Our study indicated that Trbp is a key regulator of myogenesis and skeletal muscle regeneration.

## Materials and Methods

### Mouse models

All experiments with mice were performed according to protocols approved by the Institutional Animal Care and Use Committees (IACUC) of Boston Children's Hospital (Protocol # 15-08-2986R). Boston Children’s Hospital has pathogen free mouse facilities with regulated hour light/dark cycles and climate control. Veterinary and animal care staff change cages and ensure the health of the mice. The facilities are AAALAC certified and have active Animal Welfare Assurance certification(AAALAC Accreditation Granted on 2/24/1992, The Animal Welfare Assurance number: A3303-01). Condition of the mice was monitored every day. For each individual experiment, at least 3 mice (n≥3) were used. Mice were euthanized by CO2 delivered from a compressed gas source. Neonatal rodents are resistant to CO2 euthanasia and were euthanized by decapitation using sharp scissors. These methods are consistent with the recommendations of the Panel on Euthanasia of the American Veterinary Medical Association. The Trbp-KO, Trbp-floxed mice, myf5-CreTG mice (Jax Lab, 007893) were described previously[4, 23].

### Cardiotoxin injury

Cardiotoxin from *Naja Mossambica mossambic*a (Sigma-Aldrich) was dissolved in sterile saline to a final concentration of 10uM. For each ~6-week old mouse, 50 ul of cardiotoxin solution was injected with a 27 Gauge needle into one tibialis anterior (TA) muscle, same volume of saline was injected into the other TA muscle as control. During cardiotoxin injection, animals were anesthetized with isofluorane and under the protocols approved by the Institutional Animal Care and Use Committee.

### Histology and immunostaining

Mouse skeletal muscle tissues were dissected out, rinsed with PBS and fixed in 4% paraformaldehyde overnight at 4°C. After dehydration through a series of ethanol baths, samples were embedded in paraffin wax as previously described [23]. Sections of 10 μm were stained with Haematoxylin and Eosin (H&E), or further fixed with pre-warmed Bouins’ solution, 55°C for 1 hour, and stained with Fast Green and Sirius Red according to the routine protocol. The stained sections were subjected to histological examination with light microscope.

### C2C12 Cell culture, transfection, and differentiation

C2C12 myoblasts were cultured in growth medium (10% FBS in DMEM) as previously described [12, 25]. Control siRNA (MISSION^®^ siRNA Universal Negative Control #1, Sigma) or Trbp siRNAs (SASI_Mm02_00315864, SASI_Mm01_00138711, Sigma) were transfected into the cells at the concentration of 100nM with Lipofectamine^®^ RNAiMAX Reagent (Life Technologies). Myogenic differentiation was induced as previously described [26]. In brief, cells were maintained in growth medium. When they reached ~90–100% confluence, cells were switched to medium containing 2% horse serum to induce differentiation. Differentiation of cells was monitored by immunostaining with MF20 antibody (Developmental Studies Hybridoma Bank, University of Iowa, Iowa City).

### Western blot

Western blot analysis was performed as previously described [23]. In brief, lysates samples were prepared from tissues or cultured cells in RIPA-based buffer, separated by SDS-PAGE gel, and electrophoretically transferred to PVDF membranes. The membrane was probed with a mouse anti-Trbp antibody (Thermo Scientific, #LF-MA0209) and the mouse MF20 antibody. Gapdh, or β-tubulin was used as loading controls (mouse anti-Gapdh EMD Millipore, MAB374 or mouse anti-β-tubulin,Sigma, T8328, respectively). Protein bands were visualized with Odyssay image system (LI-COR).

### qRT-PCR (Quantitative RT-PCR)

RNA was purified using Trizol reagent. cDNA synthesis was performed using random primers and MMLV reverse transcriptase (Invitrogen) in 20 μl reaction system. Quantitative PCR was performed with Sybr Green chemistry, using GAPDH as the endogenous control. miRNA cDNA synthesis was performed using the TaqMan^®^ MicroRNA Reverse Transcription Kit. The level of miRNAs was assayed using Taqman miRNA assay kit. U6 snRNA was used as internal control.

## Result

### Genetic deletion of Trbp results in growth retardation and reduced skeletal muscle tissue mass

**Fig 1:**
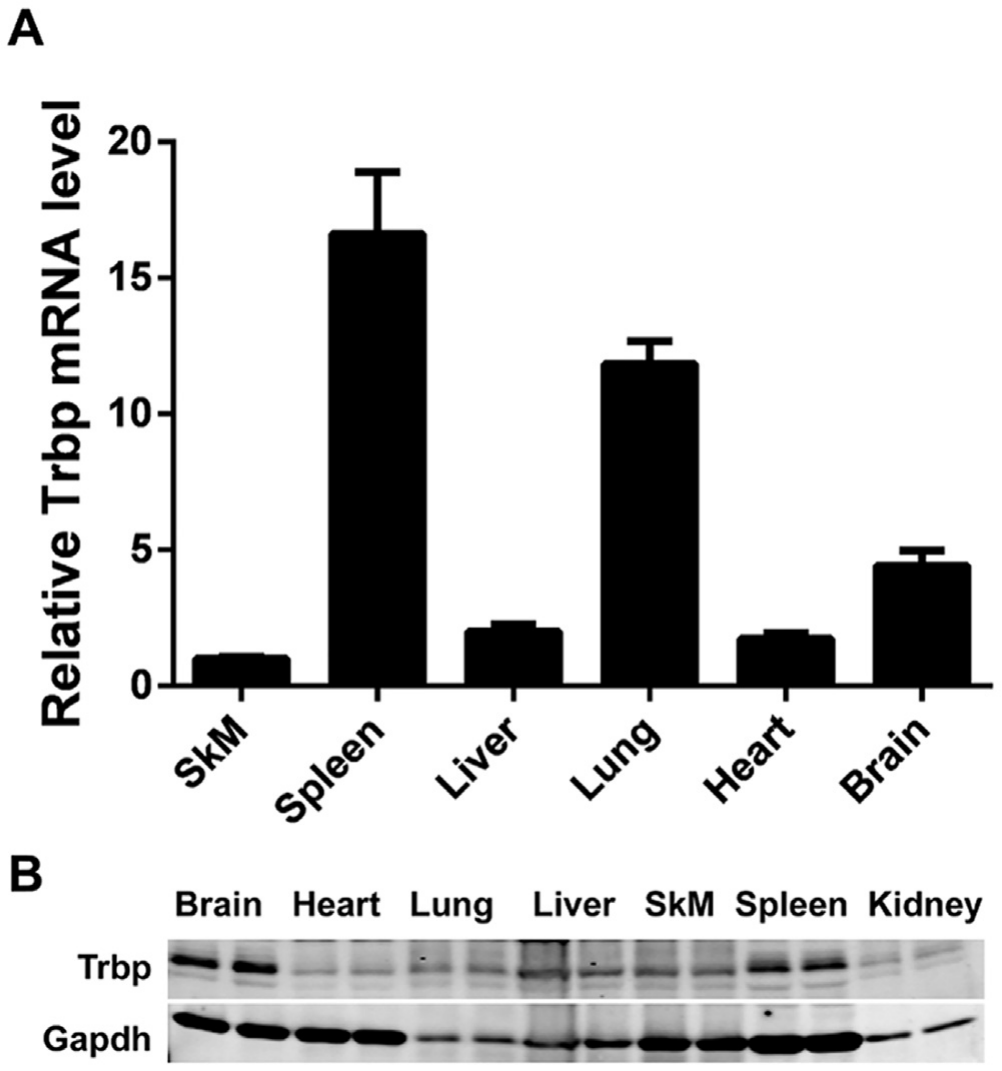
Expression of Trbp in mouse tissues. (A) Expression of Trbp mRNAs in skeletal muscle (SkM), spleen, liver, lung, heart and brain tissues of adult mice as detected by qPCR assays (n = 3); (B) Western blots detecting the expression of Trbp proteins in brain, heart, lung, liver, skeletal muscle (SkM), spleen and kidney tissues of adult mice.

We investigated the distribution of Trbp expression in adult mice. We first examined the expression levels of Trbp mRNA from different tissues and organs of 4 months old mice. Trbp mRNA is widely expressed in adult mice, consistent with previous reports [27]. In particular, we detected high levels of Trbp expression in spleen and lung. The expression of Trbp in skeletal muscle is comparable with that of heart and liver (Fig 1A). We also examined the level of Trbp protein using western blot, further confirmed that this RNA-Binding Protein (RBP) is expressed in various tissues (Fig 1B).

**Fig 2:**
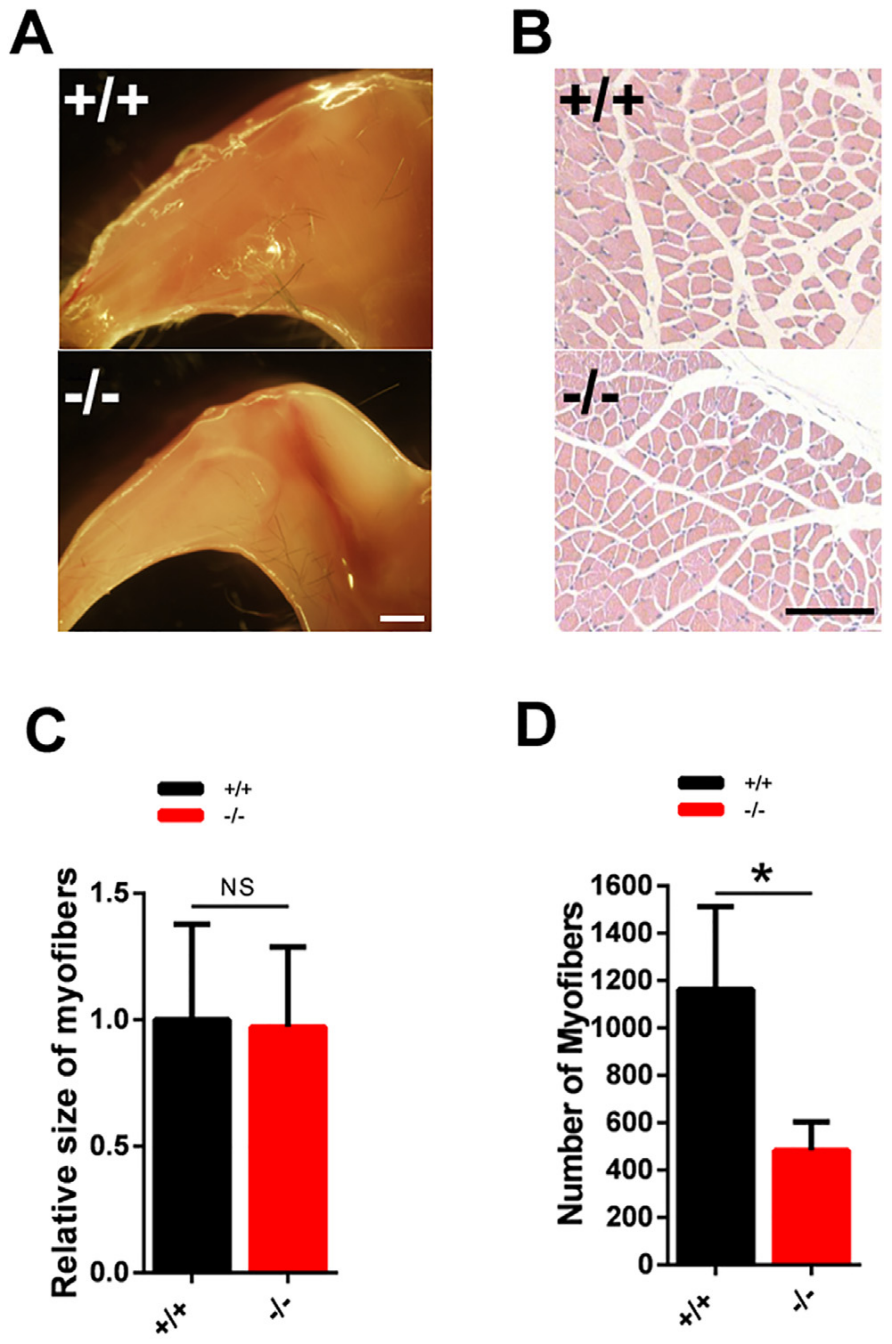
Skeletal muscle phenotype in Trbp mutant and control mice. (A) Gross morphology of the hind legs from 2-week old mice. Scale Bar = 2mm; (B) Hematoxylin and eosin (H&E) staining of transverse section of Tibias anterior (TA) muscle of 2-week-old mice. Scale Bar = 100um; (C) Quantification of myofiber size. (D) Quantification of myofiber numbers of TA muscle. At least 3 mice for each genotype were quantified and data are presented as Mean ± SEM. NS, not significant. +/+, wild type; -/-, Trbp mutant.

We, and others, have previously reported that global inactivation of Trbp in mice resulted in postnatal lethality [23, 24]. Homozygous Trbp mutant mice exhibit smaller body size, compared with their wild type littermates. Since skeletal muscle makes up a substantial part of the body weight, we asked if the reduced body size is associated with an alteration of skeletal muscle in Trbp mutant mice. Indeed, we found a substantial reduction of muscle mass in Trbp mutant mice (Fig 2A). We performed histologic analysis of the tibialis anterior (TA) muscle and we did not observe any difference in the morphology of myofiber between mutant and control mice (Fig 2B). Quantitative measurement indicated that the size of myofibers in Trbp mutant mice is comparable to that of wild type mice (Fig 2B and 2C). Instead, Trbp mutant mice exhibit a decrease in the total number of myofibers in the TA muscle (Fig 2D).

### Trbp is dispensable for normal skeletal muscle development

**Fig 3:**
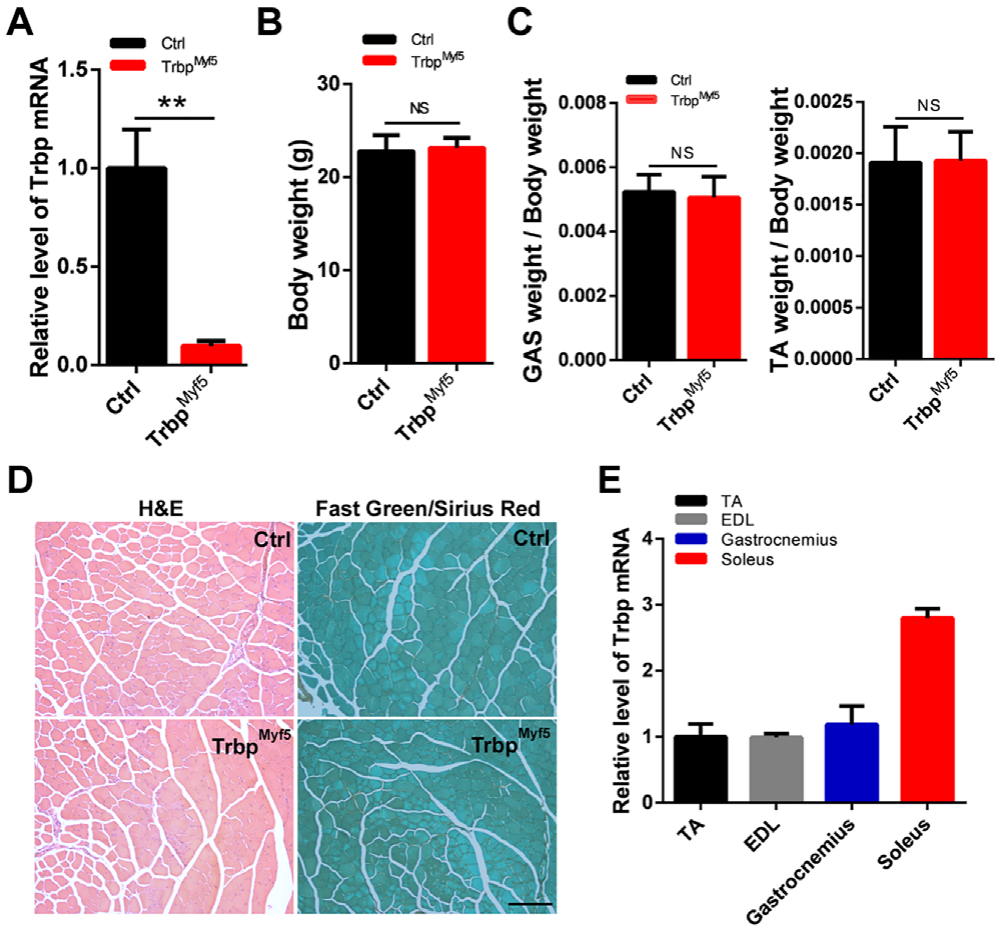
Phenotypic characterization of skeletal muscle-specific Trbp mutant mice. (A) qRT-PCR of Trbp mRNA levels in TA muscle tissues of 4-week control and mutant mice (n = 3); (B) Body weight of 4-week old control and mutant mice (n = 5–6); (C) GAS and TA muscle weight of 7-week old control and mutant mice (n = 5–6); (D) H&E and Fast green/Sirius red staining of transverse sections of TA muscle from 7-week-old control and mutant mice. Scale Bar = 200um; (E) qRT-PCR of Trbp mRNA levels in TA, EDL, Gastrocnemius and Soleus muscle tissues of 4-week old wild type mice (n = 3).

We asked if the phenotype observed in conventional mutants is due to loss of the Trbp activity in skeletal muscle *per se*. Taking advantage of the Trbp-floxed (Trbp^fl/fl^) allele that we have established previously, we inactivated Trbp in skeletal muscle by crossing the Trbp^fl/fl^ with Myf5-Cre mice, in which the muscle-specific Myf5 promoter was used to drive the expression of Cre recombinease in skeletal muscle [4]. The resultant Trbp^fl/fl^::Myf5-Cre mice (thereafter called Trbp^Myf5^) are viable, fertile, without overt abnormality when compared with their wild type littermates. We confirmed that Trbp expression was abolished in the skeletal muscle of Trbp^Myf5^ mice (Fig 3A). In sharp contrast to conventional Trbp mutant mice, which display substantial reduction in body size and weight, the body weight of the conditional Trbp^Myf5^ mice is similar to their control mice (Fig 3B). We measured the ratios of gastrocnemius (GAS)/body weight and tibialis anterior (TA)/body weight and found no difference between Trbp^Myf5^ and control mice (Fig 3C). Histologic analyses incorporating Haematoxylin & Eosin (H&E) and Sirius Red/Fast Green staining of the TA muscle revealed no difference between Trbp^Myf5^ and control mice (Fig 3D).

Our previous study has identified Trbp as a key regulator of slow- and fast-twitch muscle gene programs in the heart [23]. Unlike cardiac muscle, skeletal muscle consists of both slow-twitch and fast-twitch myofibers that are enriched within different types of muscle [28]. We analyzed the expression level of Trbp in multiple skeletal muscle tissues. We found that Trbp is expressed in all skeletal muscle subtypes tested, including the extensor digitorum longus (EDL), GAS and TA muscles with highest expression detected in the soleus muscle (Fig 3E).

**Fig 4:**
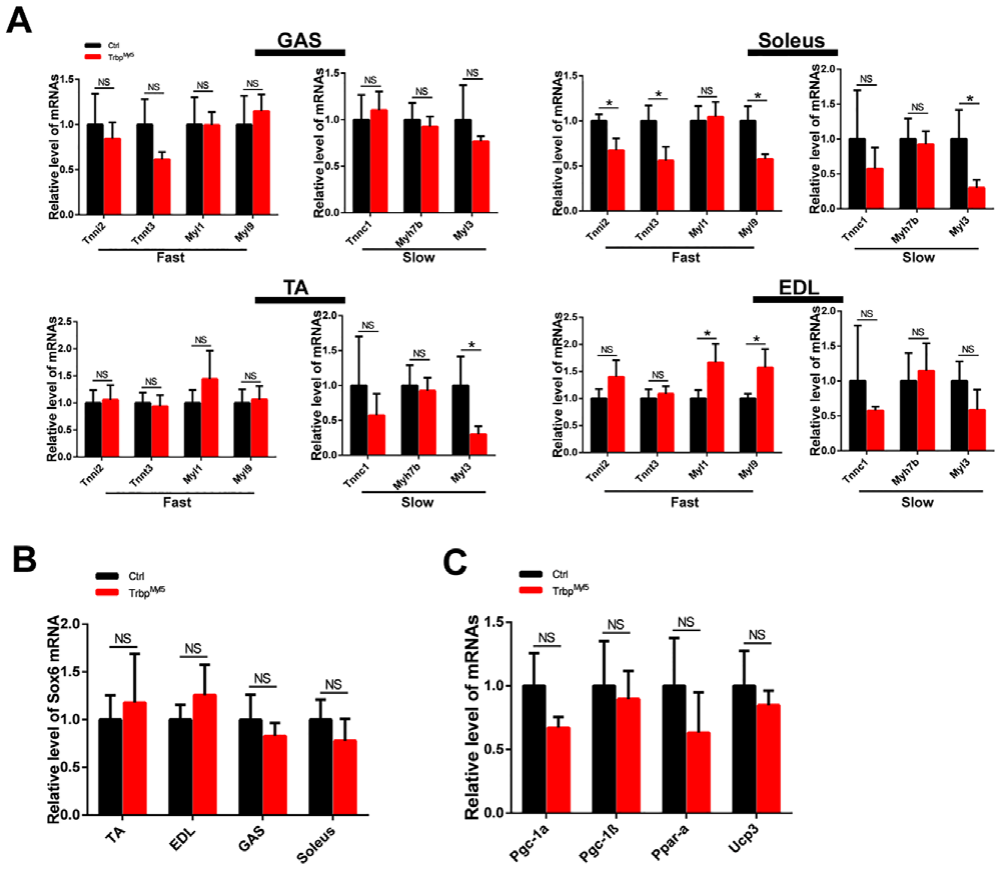
Fast- and slow- twitch myofiber gene expression in skeletal muscle-specific Trbp mutant mice. (A) qRT-PCR analysis of fast- and slow- twitch myofiber genes in GAS, Soleus, TA and EDL muscle tissues of 4-week old control and mutant mice (n = 3); (B) qRT-PCR of Sox6 mRNA levels in TA, EDL, GAS and Soleus muscle tissues of 4-week old control and mutant mice (n = 3); (C) qRT-PCR analysis of metabolic markers in skeletal muscle of 4-week old control and mutant mice (n = 3). Data are presented as Mean ± SEM. Ctrl, control; Trbp^Myf5^, Trbp^f/f^::Myf5-Cre. EDL, extensor digitorum longus; GAS, gastrocnemius; TA, tibias anterior. NS, not significant. *, P<0.05.

Next, we examined the expression of fast- and slow-twitch myofiber genes in different types of muscle tissues of Trbp^Myf5^ and control mice. Consistent with previous reports, the expression of slow-twitch myofiber genes is relatively higher in soleus muscle [28]. Unexpectedly, loss of Trbp in skeletal muscle did not shift the expression of slow/fast twitch gene program. Instead, we only observed very mild, often non-statistically significant change in the expression of these genes (Fig 4A). This observation is in contradistinction to what we observed in the heart. As revealed in our previous study, Sox6 is a key factor functioning downstream of Trbp to modulate the expression of fast- and slow-twitch myofiber genes in the heart [23, 29]. We analyzed Sox6 mRNA level in the skeletal muscle of Trbp^Myf5^ mice, yet found it is not altered there (Fig 4B). Thus, our results suggest that the regulatory effects of Trbp on Sox6 and its downstream fast/slow gene program are cardiac-specific.

Next, we examined metabolic markers in the skeletal muscle samples of Trbp^Myf5^ and control mice, given that Trbp has been reported to participate in the regulation of liver immunometabolism [22]. As shown in Fig 4C, inactivation of Trbp did not alter the expression of these molecular markers, including PGC-1α, PGC-1β, PPAR-α and UCP-3, in the skeletal muscle.

Together, we found that skeletal muscle specific Trbp mutant mice are phenotypically normal under physiological conditions, suggesting that Trbp is dispensable for skeletal muscle development and function. In contradistinction to what we observed in the heart, Trbp affects neither Sox6 expression nor the fast-/slow- twitch myofiber gene program in skeletal muscle. Our study indicated that the regulatory effects and function roles of Trbp are highly dependent on the biologic context.

### Trbp is required for skeletal muscle regeneration

**Fig 5:**
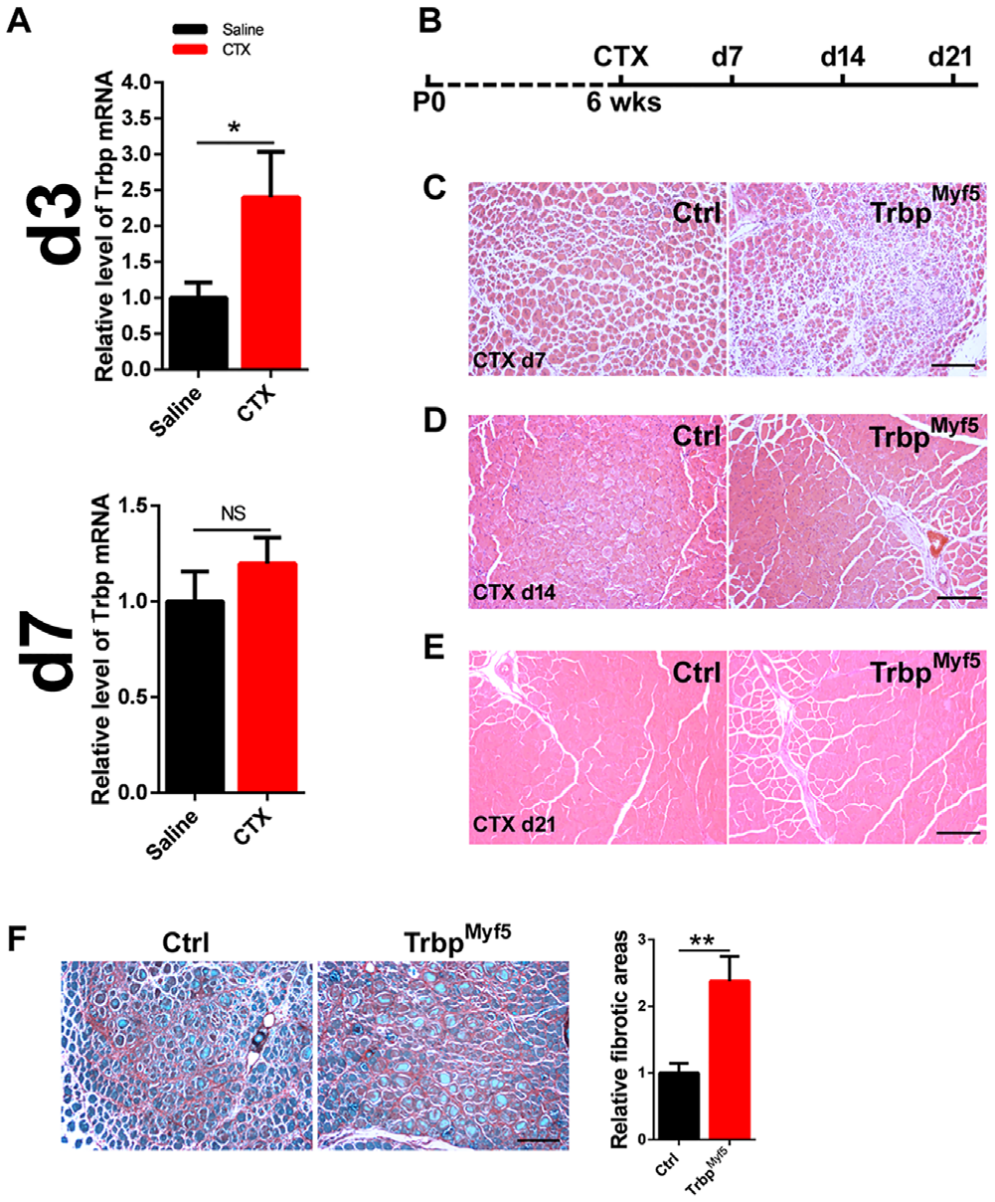
Function of Trbp in skeletal muscle regeneration. (A) qRT-PCR of Trbp mRNA levels in TA muscle tissues 3 days (d3) and 7 days (d7) after saline or CTX injection (n = 3). Data are presented as Mean ± SEM. NS, not significant. **, P<0.01; (B) Schematic of the CTX injection and the experimental procedure. (C, D, E) H&E staining of transverse section of TA muscle of control and mutant mice 7 days (C, CTX d7), 14 days (D, CTX d14), and 21 days (E, CTX d21) after CTX injection. Scale bar = 200um; (F) Fast green/Sirius red staining of transverse sections of TA muscle of control and mutant mice 7 days after CTX injection. Quantification of relative fibrotic areas was shown in bar graph (Right). At least 3 mice for each genotype were quantified and data are presented as Mean ± SEM. **, P<0.01. Ctrl, control; Trbp^Myf5^, Trbp^f/f^::Myf5-Cre. CTX, Cardiotoxin.

Next, we asked if Trbp plays a regulatory role during skeletal muscle regeneration. We used a well-established cardiotoxin (CTX)-induced muscle injury model [3]. Six week-old adult mice were injected with CTX in their TA muscle. We then examined muscle degeneration and regeneration at different time points post injury. We first analyzed the expression of Trbp itself in CTX-injected muscle tissue. Intriguingly, qRT-PCR assay revealed elevated levels of Trbp mRNA in the muscle tissue three days after CTX injection; however, such increase was markedly diminished four days later (Fig 5A). The altered expression pattern of Trbp suggested that it may participate in muscle injury responses and/or regeneration. To test this hypothesis, we treated Trbp^Myf5^ and control mice with CTX, and monitored the muscle regeneration at different time points after injury (Fig 5B). At day 7 after CTX treatment, we observed newly regenerated myofibers, which contain centrally located nuclei, are smaller in Trbp^Myf5^ mice than those of controls (Fig 5C). By 14 days after injury, skeletal muscle of both Trbp^Myf5^ and control mice are regenerated to a comparable level (Fig 5D). By 21 days after injury, regenerated skeletal muscles in both genotypes are indistinguishable (Fig 5E). Interestingly, Trbp^Myf5^ muscle exhibited increased fibrosis upon CTX-injury, when compared with the wild type controls (Fig 5F), further supporting the view of impaired muscle regeneration in these mice. Together, our observations suggested that Trbp is required for normal muscle regeneration.

### Knockdown of Trbp represses the expression of myogenic miRNAs and inhibits myogenesis in C2C12 myoblasts

**Fig 6:**
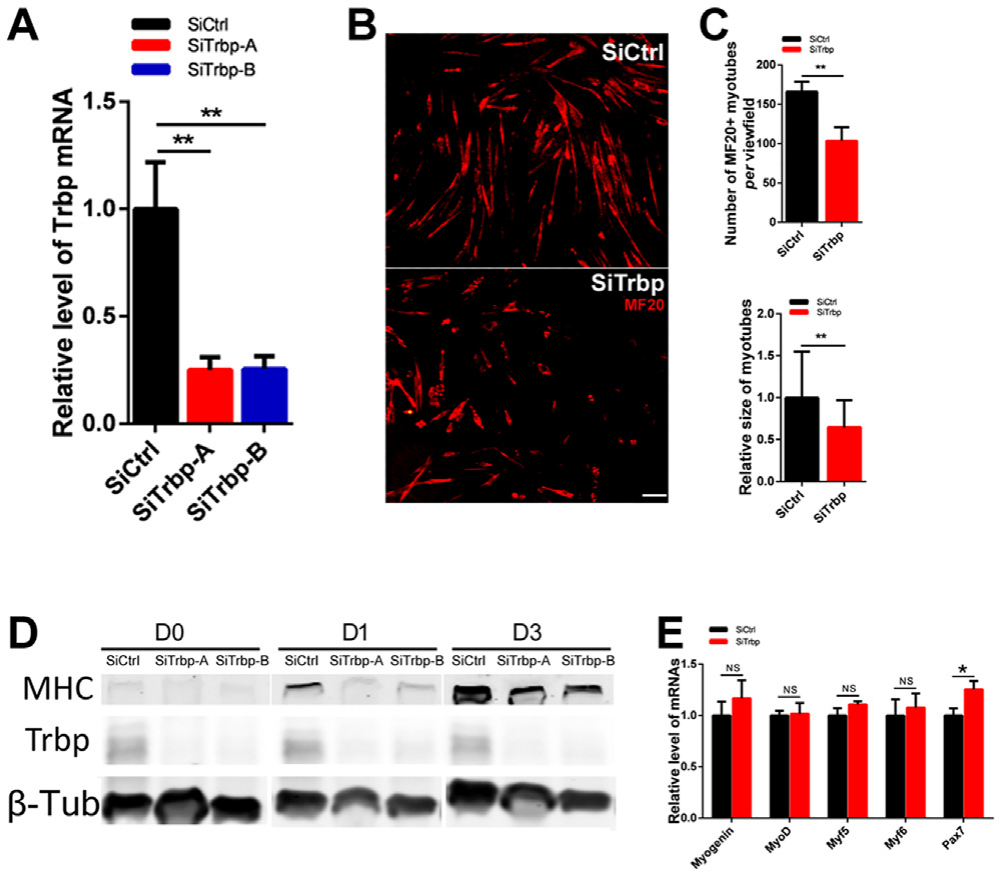
Trbp is required for C2C12 myoblast differentiation. (A) qRT-PCR of Trbp mRNA levels in siRNA-treated C2C12 cells (n = 3); (B) Representative images of C2C12 cells at differentiation day 3, stained with MF20 antibody. Scale bar = 80um; (C) Quantification of MF20 positive cell numbers (upper) and size of myotubes (bottom). More than 3 random view fields for each treatment were assayed. (D) Expression of myosin heavy chain (MHC) and Trbp proteins in SiCtrl or SiTrbp-treated C2C12 cells at differentiation day 0 (D0), day1(D1) and day3 (D3). (E) qRT-PCR analysis of myogenic transcription factors in SiCtrl or SiTrbp-treated C2C12 cells at D0 (n = 3). Data are presented as Mean ± SEM. NS, not significant. *, P<0.05. **, P<0.01.

Next, we utilized the *in vitro* myogenesis model to test the effects of Trbp on myoblast differentiation. C2C12 myoblasts were transfected with siRNAs targeting Trbp (SiTrbp). Efficient knockdown of Trbp by two independent siRNAs was confirmed with qRT-PCR and western blot (Fig 6A and 6D). 48 hours after transfection, cells were switched to low-serum medium to induce myogenic differentiation. At differentiation day 3 (D3), the majority of muscle cells treated with the control scramble siRNA-treated (SiCtrl) differentiated into multi-nucleated myotubes. However, far fewer myotubes were formed in cells treated with Trbp siRNAs (Fig 6B). Quantitative measurement confirmed significant reduced myotube formation in SiTrbp samples (Fig 6C). Using western blot, we examined the expression levels of myosin heavy chain (MHC), a terminal myogenic differentiation marker gene. As shown in Fig 6D, knockdown of Trbp substantially reduced the levels of MHC proteins during differentiation at D1 and D3. Together, our data suggests that knockdown of Trbp represses myogenesis of C2C12 myoblasts.

To better understand how Trbp regulates myocyte differentiation, we asked if the expression of key myogenic transcription factors was altered by Trbp knockdown. The mRNA levels of Myogenin, myoD, myf5 and Myf6 were assayed with qRT-PCR and found unaffected by Trbp knockdown (Fig 6E). Interestingly, we detected slight but consistent increase of Pax7 mRNA level in siTrbp-treated cells (Fig 6E). Pax7, an important transcription factor essential for skeletal muscle satellite cell function, was previously reported as a repressor of myogenic differentiation [11, 30]. The elevated level of Pax7 in Trbp knockdown myoblasts therefore may attribute to the regulatory effects of Trbp on myoblast differentiation.

### Trbp modulates the expression of myogenic miRNAs

**Fig 7:**
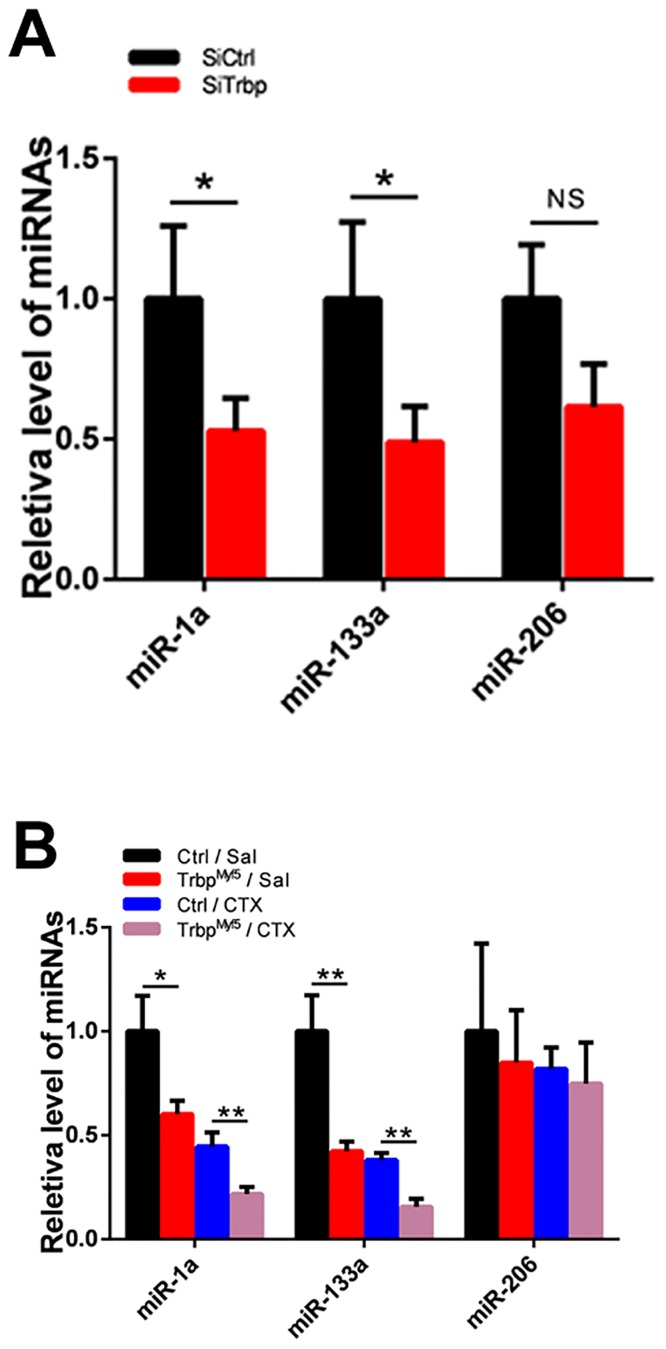
Effects of Trbp on the expression of myogenic miRNAs. (A) qRT-PCR analysis of myogenic miRNAs in SiCtrl or SiTrbp-treated C2C12 cells at D1 (n = 3). (B) qRT-PCR analysis of myogenic miRNAs in TA muscles of control and Trbp^Myf5^ mice 7 days after saline (Sal) or cardiotoxin (CTX) injection (n = 3). Data are presented as Mean ± SEM. NS, not significant. *, P<0.05. **, P<0.01.

Trbp is a key factor regulating the processing and function of miRNAs [14, 15, 23]. Numerous miRNAs have been implicated in myoblast differentiation [6, 7, 10, 11]. We hypothesized that Trbp regulates myogenic differentiation by modulating the expression of miRNAs. We analyzed the expression level of several muscle enriched miRNAs, including miR-1a, miR-133a, and miR-206, in siRNA-treated C2C12 cells. As expected, we observed that the levels of miR-1a and miR-133a were significantly reduced when Trbp was knockdown (Fig 7A). We also found reduced expression of miR-206 in Trbp knockdown sample, though it did not achieve statistical significance. Intriguingly, miR-1a has been reported to promote myogenesis by repressing Pax7, which was modestly upregulated in SiTrbp-treated cells (Fig 6E). Similarly, we examined the expression of these miRNAs in the skeletal muscle of Trbp^Myf5^ and control mice. Consistent with the results in C2C12 cells *in vitro*, we observed that the expression levels of miR-1a and miR-133a were significantly reduced in the muscle of Trbp^Myf5^ mice *in vivo* (Fig 7B). Finally, we asked whether downregulation of the expression of these myogenic miRNAs is responsive to muscle injury. As shown in Fig 7B, we found that while CTX-induced muscle injury resulted in reduced level of miR-1a and miR-133a (Fig 7B), loss of Trbp further reduced the expression levels of miR-1a and miR-133a in Trbp^Myf5^ muscle (Fig 7B). Together, our data indicates that Trbp may regulate myogenesis through modulating the expression levels and function of muscle miRNAs.

## Discussion

The myogenic process is important for muscle development, homeostasis and regeneration. Multiple muscle diseases have been found associated with defects of the myogenic regulatory program. In the face of extensive investigation, the mechanisms of myoblast differentiation remain unclear. The functional roles of muscle-enriched miR-1a and miR-133a, and their targets in myogenesis have been reported previously [7, 10, 11]. However, relatively less is known about how the expression and activity of these miRNAs are regulated. In this study, we identified Trbp as a key regulator of myogenic miRNAs, miR-1a and miR-133a, in skeletal muscle cells. Expression of miR-1a and miR-133a was dramatically down-regulated both *in vitro* and *in vivo*, when Trbp was inactivated. Inhibition of Trbp results in the delay of muscle regeneration upon injury; In addition, knockdown of Trbp represses C2C12 myoblasts differentiation *in vitro*. Together, our study established a unique role of Trbp in modulating myogenesis and muscle regeneration.

In our previous study, which focused on cardiac muscle, we found that inactivation of Trbp leads to an elevated expression of Sox6, resulting in a slow- to fast-twitch myofiber gene program shift. One of the miRNAs that mediates the function of Trbp in the heart is the cardiac-specific miR-208a [23]. The regulatory role of Sox6 in controlling slow- and fast-twitch gene expression is conserved in both cardiac and skeletal muscle [29, 31]. Surprisingly, in skeletal muscle specific Trbp mutant mice, we observed neither upregulation of Sox6 nor alteration of slow- and fast-twitch gene expression. Our data suggests that the regulatory effects of Trbp on Sox6 expression may be cardiac-specific.

The muscle-enriched miR-1a is expressed in both cardiac and skeletal muscles. Here, we found that Trbp is required for the normal expression and function of miR-1a in skeletal muscle. However, in our previous study, cardiac-specific knockout of Trbp did not significantly alter the level of miR-1a in the neonatal heart [23]. Thus, the regulation of miRNA expression by Trbp and its substrate specificity appear to be context-dependent, as has been implicated in numerous previous reports. There are often inconsistencies among different studies regarding the effect of Trbp on the processing of individual miRNAs under different conditions [14, 15, 17, 20]. For example, the study by Haase et al. showed that the extracts from Trbp-KD (knockdown) cells was deficient in pre-Let-7 processing in a cell-free system, while the steady level of Let-7 was not significantly altered in these cells [15]. Our studies strongly suggest that the specificity of Trbp on miRNA expression is context-dependent. We further postulate the existence of cardiac- and/or skeletal muscle- specific cofactors that reshape the substrate specificity of Trbp and confer the regulatory effects of Trbp on muscle miRNA expression.

Pax7 acts as a key regulator of myogenesis by maintaining the “stemness” of the myoblasts and repressing the differentiation process. Pax7 has been demonstrated as a downstream target of miR-1a (and miR-206) [11]. In our study, the level of miR-1a was drastically reduced when Trbp was inactivated. Consistently, we observed an upregulation of Pax7 in SiTrbp-treated C2C12 myoblasts, which is correlated with the down-regulation of miR-1a. We also tested the expression level of other previously identified targets of miR-1a and miR-133a in Trbp knockdown myoblasts, however, we did not detect the alteration of the expression of these targets. We speculate that the regulatory effects of Trbp on C2C12 differentiation are at least partially attributable to the up-regulation of Pax7. However, we cannot formally exclude the potential involvement of other unknown regulators, including miRNAs and protein coding genes, which are regulated by Trbp or miRNAs in skeletal muscle. Many additional miRNAs, including miR-98 and miR-631, are involved in myogenesis, as revealed by recent studies [32, 33]. Given the key roles of Trbp in miRNA processing, it will be interesting to test if these miRNAs also participate in Trbp-regulated skeletal muscle differentiation in the future.

Our study showed that conventional knockout of Trbp results in reduced skeletal muscle size. However, the gross morphology of muscle tissue in skeletal muscle specific Trbp mutant mice appears normal. Our data therefore suggests that the muscle defects in conventional Trbp null mice are not due to the loss of Trbp in skeletal muscle *per se*; instead, Trbp activity in other tissues/organs is required for the normal development and growth of the whole body. Consistent with this idea, we found that the weights of many organs, including liver, spleen, kidney and heart are significantly decreased in conventional Trbp mutant mice, and the reduction appears to be proportional to the decrease of body weight. It will be interesting for future studies to identify the organ(s) in which Trbp critically modulates global growth and development.

## Supporting Information

S1 Table Sequences of qRT-PCR primers. (PDF)
