# Supplementary material for: Trbp Is Required for Differentiation of Myoblasts and Normal Regeneration of Skeletal Muscle
Source: PLoS One. 2016 May 9;11(5):e0155349. doi: 10.1371/journal.pone.0155349 (PMC4861269; doi:10.1371/journal.pone.0155349)
Supplement: S1 Table — (PDF) [file pone.0155349.s001.pdf]

Supporting Information

S1 Table. Sequences of qRT-PCR primers

Gene	Primer	Sequence
**Trbp, TAR DNA binding protein**	Trbp-qF	aggagggaatgagtgaagagg
	Trbp-qR	gaaggctgatcggggtct
**Sox6, SRY-box 6**	Sox6-qF	ttacaaccacagacagattgagc
	Sox6-qR	tggctgtggagttgatgg
**Tnni2, troponin I2, fast skeletal type**	Tnni2-qF	gagatgaggagaagcgcaac
	Tnni2-qR	cgctatctggagcatcacac
**Tnnt3, troponin T3, fast skeletal type**	Tnnt3-qF	ttgaccaagcccagaagc
	Tnnt3-qR	gcagtgcacctctctgctct
**Myl1, myosin, light polypeptide 1**	Myl1-qF	caatggctgcatcaactatga
	Myl1-qR	gccataggtgttcttgaactgg
**Myl9, myosin, light polypeptide 9, regulatory**	Myl9-qF	gataaggaggacctgcacga
	Myl9-qR	gccctccagatactcgtctg
**Tnnc1, troponin C, cardiac/slow skeletal**	Tnnc1-qF	cgacagcaaagggaagtctg
	Tnnc1-qR	tgtagccatcagcgtttttg
**Myh7b, myosin, heavy chain 7B, cardiac muscle, beta**	Myh7b-qF	gcacggagctcaagaaagac
	Myh7b-qR	gccatctcttctgtcaggttct
**Myl3, myosin, light polypeptide 3**	Myl3-qF	aagaaggatgatgccaaagc
	Myl3-qR	gctcaggctctggtgcag
**Pgc1-α, PPARG coactivator 1 alpha**	Pgc1-α-qF	actgacggcctaactccaccca
	Pgc1-α-qR	actcggattgctccggccct
**Pgc1-β , PPARG coactivator 1 beta**	Pgc1-β-qF	tcctgtaaaagcccggagtat
	Pgc1-β-qR	gctctggtaggggcagtga
**Ppar-α, peroxisome proliferator activated receptor alpha**	Ppar-α-qF	ctgagaccctcggggaac
	Ppar-α-qR	aaacgtcagttcacagggaag
**Ucp3, uncoupling protein 3**	Ucp3-qF	ggatgcctacagaaccatcg
	Ucp3-qR	ttgtgatgttgggccaagt
**Myog, myogenin**	Myog-qF	agcgcaggctcaagaaagtgaatg
	Myog-qF	ctgtaggcgctcaatgtactggat
**MyoD, myogenic differentiation 1 (MyoD1)**	MyoD-qF	cgccactccgggacatag
	MyoD-qR	gaagtcgtctgctgtctcaaagg
**Myf5, myogenic factor 5**	Myf5-qF	cagccccacctccaactg
	Myf5-qR	gggaccagacagggctgtta
**Myf6, myogenic factor 6**	Myf6-qF	atcagctacattgagcgtctaca
	Myf6-qR	cctggaatgatccgaaacacttg
**Pax7, paired box 7**	Pax7-qF	ggcacagaggaccaagctc
	Pax7-qR	gcacgccggttactgaac
